# Coming out in the university workplace: a case study of LGBTQ + staff visibility

**DOI:** 10.1007/s10734-022-00884-y

**Published:** 2022-06-14

**Authors:** Catherine Lee

**Affiliations:** Cambridge, UK

**Keywords:** LGBTQ +, Diversity, Inclusion, Visibility, Coming out, Workplace, Staff

## Abstract

This article explores the issue of workplace visibility and signs and symbols of LGBTQ + identity in a UK university. A poststructuralist Butlerian theoretical framework underpins this article. Sexual and gender identities are understood as multiple and fragmented, and constructed in relation to others and within the systems of power and knowledge that exist in universities and society more widely. An anonymous survey and focus group discussions were conducted with LGBTQ + staff in a higher education institution in England awarded university status in 1992. Results showed that staff felt relatively comfortable coming out to their peer-groups in the workplace but were less confident in coming out to students. Signs and symbols of LGBTQ + identities were fundamentally important to LGBTQ + staff members in helping them feel safe in the workplace and indicating to LGBTQ + students that they were potentially a source of support. The visibility of LGBTQ + senior leaders was important in empowering staff to believe that they too might progress within the university.

## Introduction

This article explores the lived experiences of lesbian, gay, bisexual, transgender and queer or questioning (LGBTQ +) academic and professional services staff in one UK university. In particular, it focusses on the issue of workplace visibility and includes coming out discourses and signs and symbols of LGBTQ + identity.

A poststructuralist Butlerian theoretical framework underpins this article. Sexual and gender identities are understood as multiple and fragmented, and constructed in relation to others and within the systems of power and knowledge that exist in universities and society more widely. The article recognises that in common with the rest of society, universities sanction identities and relationships that conform to the norms and values of heterosexuality, and the gender norms of male masculinity and female femininity but in doing so leave those existing outside these norms potentially excluded or othered. Power is understood not to operate simply in one direction however. Often the ways in which identity discourses are configured in the university workplace allow for quite powerful resistances (Epstein et. al. 2003). Coming out in the university workplace through a declarative statement (Khayatt, 1997) or through signs and symbols of LGBTQ + visibility, disrupts or queers the cis and heteronormative university workplace, creating LGBTQ + role models who permit other LGBTQ + university stakeholders to thrive within spaces that are diverse and inclusive.

An anonymous survey of LGBTQ + staff was conducted in June 2021 with colleagues from academic and professional services in a higher education institution in England awarded university status in 1992. Emerging themes and issues from the survey were investigated further during two follow-up focus groups. Results showed that staff felt relatively comfortable coming out to their peer-groups in the workplace, but were far less confident in coming out to students. Signs and symbols of LGBTQ + identities were fundamentally important to LGBTQ + staff members. When they saw symbols such as Stonewall Champion accreditation or rainbow flags on campus, this helped them feel safe in the workplace. When LGBTQ + staff members adopted symbols themselves such as Pride emojis or rainbow lanyards, this helped indicate to LGBTQ + students that they were potentially a source of support. LGBTQ + visibility was also important to confidence in career-development. When LGBTQ + staff saw LGBTQ + colleagues in senior leadership positions, they felt empowered and believed that they too might progress within the university.

This article begins with a review of the literature before presenting the conceptual framework underpinning the article. The findings of the survey and focus groups are presented and analysed before the salient issues are revisited in a conclusion.

## Literature review

Universities typically regard themselves as safe and inclusive spaces for those othered in the rest of society (Coley & Das, 2020). University is often the place in which young people, living away from the family home for the first time, come out as LGBTQ + . Despite increasing numbers of LGBTQ + staff in the university workplace (HESA, 2020), their voices are marginalised and under-researched. Coming out, the process of declaring one’s sexual or gender identity to others is, in the university workplace, complex to negotiate and takes place multiple times over the professional lifetime of university staff (Bochenek & Brown, 2001). There are personal, political and pedagogical benefits to coming out in the university workplace, and it is widely seen as empowering for university academics in particular, as it facilitates authenticity in the classroom (Yost & Smith, 2014). Authenticity is according to Sparrowe (2005) a person’s self-awareness and embodiment of their fundamental values and purpose. It emerges in relation to interactions with others and relies on self-awareness and self-regulation and needs validation from others. When a university staff member comes out in the workplace, they provide LGBTQ + students and colleagues with role models and trouble the heterosexual assumption.

It is important to note that the university workplace experiences of professional services staff differ to those of their academic counterparts. Professional services staff may or may not be student facing and may interact with students in a host of capacities, including admissions, student support, welfare and pastoral care. The UK’s Higher Education Statistics Agency (HESA) in 2012 changed the descriptors of those previous known as ‘non-academic professionals’, renaming the most senior of them ‘higher education professionals’. The reclassification signalled an expansion of professional services roles to include student and staff development, research impact, global outreach and academic research. According to Baltaru (2019) Higher level university administrators enjoy a high degree of professionalisation and expansion unlike other non-academic personnel, but notes that this is not extended to those in lower technical and manual occupations. It may be argued that there is now more to separate higher and lower graded professional services colleagues in universities than there is to distinguish academic and professional services colleagues on similar grades. Baltaru (2019) calls for an in-depth understanding of how the broader cultural environment in universities shapes local dynamics of administrative growth. By including professional services colleagues in this research, it is hoped that this article can make a modest contribution to the understanding of their inclusion in the university workplace.

### Coming out

There are ‘Coming Out Day’ initiatives in many western countries including the UK. Coming Out days are rooted in queer and feminist theoretical perspectives and encourage LGBTQ + people to come out in the workplace or to family and friends. The principles of the Coming Out Day, which originated in the USA in 1988, are that homophobia and heteronormative discourses thrive in an atmosphere of silence and ignorance, and that once people know that they have loved ones or valued colleagues who are LGBTQ + , they are less likely to maintain homophobic or transphobic views. Rasmussen (2004) notes that educational researchers are perturbed by the coming out imperative and points to limitations of this discourse in diverse educational contexts. Coming out in the university workplace allows those who feel able to do so to become beacons of equality, diversity and inclusivity, but, according to Rasmussen, leaves those who feel unable to come out as a failure or somehow less honest and open. Kehily (1999) warns that there remains ‘the ever present danger that coming out will change the way others think about you’ (p.148) and notes that such a declarative statement is not retractable. The risks for some academics and professional services staff in coming out in the university workplace cannot be underestimated. Atkinson (2002) states that fears of encountering a negative reaction or of being subject to name calling, graffiti, harassment or even physical assault are very real. Henderson (2019) suggests that the LGBTQ + educator has two realities or options; become a role model for students and risk homophobia from students or colleagues who have conservative or religious views, or embody and navigate a fragmented workplace identity where the management of incongruent personal and professional identities has over time the potential to lead to poor health and wellbeing (Meyer, 2003).

### Authenticity

Fletcher and Everly (2021), researching the life satisfaction of LGBTQ + employees, explored the roles of disclosure and authenticity at work. Their results show that the perception of LGBTQ + supportive practices in the workplace are directly linked with rates of disclosure, perceptions of authenticity at work, and are indirectly related to employee workplace and life satisfaction. Employees in the UK have for a decade enjoyed workplace protection with sexual orientation and gender reassignment two of nine protected characteristics in the Equality Act (2010). There is evidence that supportive LGBTQ + policies and practices in the workplace are linked with positive work attitudes, reduced psychological strain, and the perception of less workplace discrimination’ (Webster et al, 2018, p.489). When LGBTQ + educators are visible as their authentic selves in the workplace, they are also shown to be more loyal to their employer, more effective in their role and more receptive to the acquisition of skills conducive to leadership (Lee, 2020).

### Don’t ask, don’t tell

Sedgwick (2007) describes the closet as a regime of regulation in LGBTQ + lives. The closet is important to heterosexual and cisgender people as it guarantees their privileges. Sedgwick posits that the disclosure of sexual or gender identity does not necessarily place the agency with the person disclosing however. The deliberate wish not to know about person’s sexuality or gender identity can be an act of power over them. Don’t Ask, Don’t Tell is an act of pseudo-tolerance in university communities that can serve to silence LGBTQ + colleagues. Most commonly associated with the Clinton administration in the USA, lesbian and gay soldiers were allowed to serve in the American armed forces as long as they did not refer to their sexual identity or betray it within their actions and physical presentation. Don’t Ask, Don’t Tell, when applied to education (DePalma & Atkinson, 2006; Thompson-Lee, 2017), refers to the absence of a safe space in the university or school, classroom or office for the LGBTQ + employee to speak their personal identity into existence. LGBTQ + identities in the university workplace are then co-constructed between the individual and their university community, and are constantly revisited and reconstructed in different contexts and through different relationships and alliances. Fric (2019) found that employees who need to conceal their sexual or gender identity at work are less likely to report discrimination but are more likely to perceive being discriminated against. Research by Lee with educators in schools (2019) supports this, with teachers who did not feel able to be out in the school workplace, more likely to experience micro-aggressions and hear homophobic language.

In her article ‘Sex and the teacher: Should we come out in class?’ Khayatt (1997) challenges the assumptions that LGBTQ + university lecturers have a duty to come out and act as role models for LGBTQ + students, positing that coming out is not necessarily helpful for either the students or the LGBTQ + academic. Khayatt interrogates the pedagogical benefit of coming out in the classroom, particularly through a ‘declarative statement’ (p.108). Khayatt believes the act of making a declarative statement serves to freeze the identity of the LGBTQ + academic in space and time, rendering them incapable of evolving further in relation to their students.

### Symbols of LGBTQ + inclusion

Universities can do a good deal to create cultures that promote equality diversity and inclusion and celebrate difference. Equality kite marks such as Stonewall Diversity Champion Awards, help signal to LGBTQ + employees that the university is a safe space in which to come out. Rainbow flags, badges and lanyards can similarly signify LGBTQ + inclusion. Calvard et al. (2020) states that LGBTQ + diversity signifiers can influence a person’s decision to come out as they indicate the presence of other LGBTQ + people and allies in the workplace. Prasad et al, (2011) concur but caution that even where an organisation has the best of intentions to create an inclusive workplace, symbols alone, especially when displayed once or twice a year during Pride and LGBTQ + History months can feel half-hearted and hollow when there is an absence of meaningful policy, activity, or inclusive practice behind the symbols. According to Calvard et al. (2020), for a university to be truly inclusive, overt symbols of LGBTQ + visibility must form part of culture and policy that engages all university stakeholders in LGBTQ + inclusive practice and genuinely celebrates diversity.

### Role models and leadership

Just as LGBTQ + academics and professional services staff can be role models to students, then LGBTQ + leaders in universities can be role models to other staff, in particular aspiring leaders. When out LGBTQ + university employees are promoted to positions of leadership, it sends a very clear signal that the university is a safe space for LGBTQ + employees and one in which their career can thrive. LGBTQ + university leaders must however, navigate complex cultural and political workplace environments whilst under the gaze of a host of different stakeholders. According to Fine (2017) to be successful, LGBTQ + leaders must present themselves in exactly the same way as their heterosexual/cis gendered peers, betraying nothing in their behaviour or communication that challenges or threatens the hetero and or cis normative status quo. Clarke (1996) refers to this as the ‘good homosexual’, that is an individual who is out in the workplace but is completely without reproach, and embodies in their workplace presentation behaviours that are read as heterosexual or cis-gendered. The conventional Western leadership trope of ambition, strength, power and assertiveness is imbued with masculinity and so it is vital for the male (and trans-male) university leader to subscribe to a model of masculinity. However the expectations of female (and trans-female) leaders are more complex to navigate, as femininity must be apparent so that they are intelligible as a woman to their followers. Fassinger (2008), states that there is an obvious and ironic double bind for some lesbian leaders because a lesbian might not be viewed as a ‘real’ woman, but as a woman she may embody non-traditional leadership. It can be challenging for LGBTQ + aspiring leaders in universities to be intelligible to potential followers and thrive within a heteronormative and cis-normative culture if there is dissonance between their embodiment of leadership and expectations of what a leader should look like. Femininity in gay men, masculinity in lesbians, or transgender and non-binary identities can hinder the promotion prospects of LGBTQ + aspiring leaders in universities (Christo, 2014). Conversely, Fletcher and Everly (2021) state that when LGBTQ + colleagues are visible to others in the workplace it stimulates within others ‘interpersonal fluency or the ability to be oneself’ (Fletcher & Everly, 2021, p.233). Fletcher and Everly argue that LGBTQ + supportive practices promote self-concept fit and fulfil important psychological needs necessary for authenticity. Markus and Wurf (1987) define self-concept as a dynamic interpretive structure that mediates most significant intrapersonal processes including social perception; choice of situation, partner, and interaction strategy; and reaction to feedback. A strong Self-concept fit with one’s surroundings promotes a strong sense of workplace and life satisfaction. The visibility of LGBTQ + staff in universities is necessarily positive then, not just for LGBTQ + stakeholders, but also for those with other protected characteristics or perceive themselves to not fit in or be othered.

There is relatively little literature on the way in which both academic and professional services staff in universities experience the university workplace. This research aims to explore the personal, social and political benefits to coming out in the university workplace. It also seeks to examine the complex way in sexual and gender identities are structured in negotiation with peers, line-managers, senior leaders, students and other stakeholders.

### Conceptual framework

This article subscribes to a poststructuralist Butlerian theoretical framework. Sexual and gender identities are understood as multiple and fragmented, and constructed in relation to others and within the systems of power and knowledge that exist in universities and society more widely. Rigid binaries of male/female, boy/girl are engineered from the earliest years of formal education right through to university and into the workplace. Binary gender identification categories of male and female become interwoven with sexuality because they are understood in relation to whom a person desires, but are the ‘performative effect of reiterative acts’ (Butler, 1990 p.33). Gender and sexual identity has then, according to Butler, ‘no ontological status apart from the acts which together form its reality’ (1990, p.136).

However, it is important to note that the concepts of authenticity and self-concept also embraced in this article sit at odds with the Butlerian stance. The essential self is problematic here but according to Munro (2005) it is possible to reform poststructuralist discourses in a way which support constant gender and sexual identities. Silvester’s psychosynthesis model (2000) for example, includes a transpersonal element yet models a sense of self which is constant and authentic. Fuss (1989) too argues that an essential self can change whilst remaining centrally anchored for the subject and their relationships. Whittle (1998) discusses the self as both subject to social construction and existing as a core which is essential and gendered. According to Whittle the core self is felt to be authentic, allowing a base for identity building and politics. Rubin (1999) too, utilises phenomenology to model an essential self, but sees the development of an essential self as ultimately being strategically motivated.

This article recognises then that sexual and gender identities are constantly evolving. They transcend the private and become intertwined with social and political discourses of power, aimed at the preservation of social institutions, such as the family, the state and education (Gray, 2010). When LGBTQ + university staff feel unable to come out in the university workplace, they perpetuate the hetero and cis normative discourses and engage in a literal silencing about LGBTQ + identities (Paechter, 2002).

## Methodology

This article aims to understand the workplace experiences of university employees identifying as LGBTQ + . As this data was collected during the COVID-19 pandemic in 2021, it should be noted that the workplace is defined both as the university campus and online spaces. The research sought to answer the following questions:

- What are the issues associated with coming out in the university workplace for staff in academic and professional services?
- What role do signs and symbols of LGBTQ + identity play the university workplace?

The project utilised an exploratory case study (Yin, 2011) within a social constructivist and interpretivist framework. Case study research promotes the understanding of complex social phenomena and can be used to develop critical thinking (Alvarez, et al., 1990). Case studies strive towards a holistic understanding of cultural systems of action (Feagin, Orum, & Sjoberg, 1990) and cultural systems of action refer to sets of interrelated activities engaged in by the participants or actors in a social situation. Whilst the case study methodology means that generalisations about all university workplaces are not appropriate, the case study approach facilitated a rich picture of the way in which the LGBTQ + university staff experienced their university workplace in a single institution.

An anonymous survey was launched as part of the university’s Pride celebrations in June 2021 and remained open for three months. It was promoted via social media, email and the university’s LGBTQ + staff network, and was open to all staff identifying as LGBTQ + . At the conclusion of the survey, participants were invited to provide an email address to indicate they were happy to take part in focus groups to discuss in detail issues and themes emerging from the survey results.

The two focus group discussions took place via Microsoft Teams and were recorded and transcribed in full by the researcher.

## Profile of participants responding to the survey

The survey received a total of 36 responses but not every question was answered by all participants. The number of respondents equates approximately to 1.8% of the university’s total workforce. This is somewhat lower than the national picture. Advance HE Equality data for 2019–2020 shows an average of 3.8% of staff in HE identifying as LGBT across UK universities.

### Gender and sexual identity

Twenty-five people described their gender identity as either cis-woman (41.9%) or cis-man (38.7%). One person identified as non-binary and one person identified as a trans-woman. A further four participants described themselves as either a man, woman or in one case as ‘x’. Twenty-five people described their sexual identity as either gay man (41.9%) or gay woman/lesbian (38.7%), and one person identified as bisexual. Four participants selected ‘other’, describing their sexual identity as ‘pan/demisexual’, ‘asexual’, ‘no fixed sexual identity’ and ‘heterosexual transphillic’. One person identified as heterosexual.

### Other protected characteristics

There was an even split between participants in the age categories 26–39, 40–49 and 50–59. One respondent was aged between 16 and 25 and another was 60 + . Three participants identified as black and minority ethnic (BAME) with another preferring not to say. Five participants stated that they had a disability, health condition or learning difference which has a substantial and long-term impact on their ability to carry out day-to-day activities, with a further one respondent preferring not to say.

### University role

Out of 31 participants, 15 worked within academic teaching and research with the rest working in professional services. No one completing the survey worked within leadership of professional services and only two were from academic and teaching leadership (middle leader roles).

### Previous experience in higher education

For 20 participants, their current university workplace was their first experience of working in the higher education sector. Sixteen had worked in other universities before moving to the case study university.

## Focus group participants

Twelve participants (four gay men and eight lesbians/gay women) expressed a willingness to take part in the focus groups which were convened as two groups consisting of six members each. Groups were arranged based on staff availability and a doodle poll was used to determine suitable dates and colleagues were invited to join the group which was most convenient for them. This resulted in three gay or queer men and three lesbians/gay women in one group and one gay man and five gay women/lesbians in the other group.

Focus group 1

**Table:** 

Identity	Role category	Student facing	Length of time at the university
Lesbian	Academic Senior Lecturer	Yes	2 years
Gay woman	Professional services partnerships	Yes	5 years
Gay woman	Academic Associate Professor	Yes	10 years
Queer man	Academic Senior Lecturer	Yes	5 years
Gay man	Professional Services Business Manager	No	8 years
Gay man	Academic Head of Department	Yes	1 year

Focus group 2

**Table:** 

Identity	Role category	Student facing	Length of time at the university
Lesbian	Academic Associate Professor	Yes	12 years
Lesbian	Academic Lecturer	Yes	1 year
Lesbian	Academic Research	Yes	2 years
Gay woman	Professional Services Library	Yes	6 months
Gay woman	Professional Services IT	No	2 years
Gay man	Professional Services Admissions	No	4 years

The focus group discussion is a qualitative approach to learning about population subgroups with respect to conscious, semi-conscious and unconscious psychological and sociocultural characteristics and processes (Basch, 1987). Breen (2006) advocates the use of focus-group discussions for the generation of new ideas formed within a social context and recognises the way in which group reflection can prompt recollection and deter or safeguard against distortion.

### Survey results coding

The survey hosting software collated the statistical data of the closed survey responses. Open-ended questions were reviewed as raw responses initially before the process of preliminary data coding began. A combination of deductive codes drawn from the literature and research questions and inductive codes generated by the data were deployed As the coding progressed the initial codes were assembled into categories while attributes and relationships between the initial codes emerged (Saldana 2021). Once patterns in the data began to take shape the patterns were utilised to help draw together a schedule of questions and prompts for the focus groups.

### Focus group coding

The focus group transcripts were coded using a grounded theory approach consisting of four stages: open coding, theoretical coding, selective coding and theoretical memos to help create a narrative around the theory. Initially, the transcripts were analysed to identify significant concepts in the data. Common topics were given the same code to indicate their common link. Some codes were attached to simple phrases or sentences whilst others were assigned to whole paragraphs. Codes included, coming out, workplace symbols of LGBTQ + inclusion, role models and leadership. Theoretical memos were used to capture and record the emerging theory, accumulating and maturing as the analysis progressed (Pace, 2012). In the second phase of grounded theory analysis, theoretical coding determined how emergent concepts related to each other (Charmaz, 2006). From here, open coding concepts were reassembled with propositions that described the relationships between each of the concepts. Selective coding followed, which entailed limiting coding to only those concepts that relate to a core explanatory concept (Charmaz 2000: 516, Glaser, 1978: 61–2). The final phase of the grounded theory analysis involved sorting the theoretical memos into an outline and that could be written into a narrative which provided the following discussion:

**Data structure**



## Results and analysis

Three major themes emerged from the survey and focus group discussion data. These were, coming out to colleagues, coming out to students and signs and symbols of LGBTQ + identity in the workplace. Each theme is explored in turn and the sexual and gender identities of participants denoted according to the descriptors they gave themselves on the survey or prior to the start of the focus group discussion.

### Coming out to colleagues

Two thirds of respondents (19) to the survey were ‘out to everyone’ in the university. Four respondents (13.3%) were out to staff only and 2 respondents (6.7%) were out to nobody at all at work but wished that they could be. Five respondents were out to some colleagues and students. In the survey free text comments, a bisexual cis woman working in student-facing professional services stated, ‘sometimes worry about coming out with some colleagues I don’t know well.’ When asked the extent to which respondents felt confident to discuss issues of LGBTQ + diversity with colleagues, a third of the respondents ‘always’ felt confident to discuss issues of LGBTQ + diversity with colleagues but the majority of respondents (15) stated that they rarely felt confident to discuss issues of LGBTQ + diversity with colleagues. Colgan (2016) suggests that is incumbent upon employers to create workplace environments that provide employees with the safe and supportive workplace environment in which to speak their personal identities into existence.

For eight of the focus group participants, their current university was their first experience of work in the higher education sector. These eight believed that moving into higher education had given each of them permission to come out fully or partially at work for the first time in their careers, suggesting in common with the findings of Ellis (2006), when compared to US universities and other sectors, UK universities are considered liberal in their views towards LGBTQ + diversity. When respondents were asked whether they were happy to talk about their personal life to others in the university workplace, just under half said they were comfortable discussing their personal life with others. 52% said they would be happy to take a partner to a university function if heterosexual colleagues took their partners. Of those reticent to speak about their private life with in the workplace, a cis, gay man in none student- facing professional services stated:

It's not anything that the university has done, I just feel that I would not really feel comfortable, perhaps due to my own overthinking. I'm not sure the LGBT visibility is high within this university, it seems to be better in the faculties, but it definitely could be better in professional services.

A lesbian academic who had joined the university from the National Health Service (NHS) made a conscious decision to come out on commencement of her university role. She stated:

I arrived having not been terribly opening my last job because I was working within the health service. I made a definite decision that I was going to be very open with my colleagues from the start.

When asked how this had gone, the academic in her fifties acknowledged that her judgment was entangled in her own ‘paranoia’ from a lifetime of hiding her sexuality at work and living as a lesbian through less tolerant times. Prior experiences of homophobia inevitably leave a legacy for those who experience it and this can result in a cautious approach in relations with others, even when the culture or climate shifts to one of greater acceptance. Lee (2019) found that 15 years after the repeal of Sect. 28, teachers who had experienced it, remained vigilant and largely closeted in their school workplaces. Similarly LGBTQ + people worried that older people may hang onto homophobic views they were able to express without challenge in previous eras. This was very much the concern of this older lesbian academic. Of coming out to her colleagues, she said,

I think one or two of the older ones were accepting of me on the outside, but slightly surprised on the inside.

Two younger lesbian academics were more relaxed about their own coming out at university and both had brought their partners to initial visits to the university, where they met their teams for the first time. This had been a deliberate act to come out in the workplace from the outset without the need for a declarative statement (Khayatt, 1997) to their new colleagues. Both colleagues and their partners had been welcomed positively by the university staff community, and this bold initial move had been seen to pay off. Both lesbians agreed they felt a strong sense of loyalty to their university and the warm welcome and positive working environment had persuaded them to want to stay long term and commit their future to the university.

According to Ragins (2004), when the workplace is perceived to be a safe haven, LGBTQ + employees in all sectors are likely to stay longer term. There is evidence however, that once settled and included in their workplace, some LGBTQ + employees may be reluctant to move away from their organisation, turning down promotion out of a fear that they not be able to come out in a new workplace. According to Ragins, career choice for LGBTQ + employees is based largely on the avoidance of negative situations at work and discrimination avoidance, rather than the pursuit of positive career development opportunities. This can mean that LGBTQ + staff in universities do not achieve promotion to positions of leadership at the same rate as their heterosexual and cis-gendered peers (Lee, 2021).

A gay woman working in a none student-facing professional services role had initially kept her sexuality secret at work, having joined the university after a long career in a male dominated uniformed service where she described herself as ‘habitually closeted’. However, when her same-sex relationship came to an abrupt end, she broke down in her university open plan office space and, for the first time in her career, found overwhelming levels of support from her colleagues.

My transition point was when my girlfriend dumped me. I made it into work and then burst into tears in front of my boss who was like Oh my goodness, what's happened? And I just sort of blurted it out to the whole office.

Like the lesbian academic from the NHS, this gay woman was scarred by experiences of homophobia or heteronormativity in her previous role. Fearful of coming out, this gay woman initially continued the façade of heterosexuality in the workplace to conform to the dominant heteronormative culture. However, as Hewlin (2009) observes, employees who deviate from their authentic self to fit in with organisational norms often experience burnout or breakdown. In a crisis due to the breakdown of her relationship, this colleague could no longer manage the intersection of her personal and professional identities and her personal crisis spilled over into the workplace.

A gay male academic had joined the university during the pandemic from elsewhere in higher education. Although previously out another university workplace, working from home had denied him appropriate opportunities to come out to his colleagues. Further, as a single man, he found coming out at work challenging. He said,

I've been here a year but because I joined in lockdown I haven’t had the social opportunities where I might have been able to come out. Also, I’m single, so I haven't really announced it in the sense that there isn’t really anything to say, when you don’t have a partner.

Here, the importance of being intelligible to others becomes apparent. This colleague was single and accessing work remotely online and so the opportunities to make himself known, or for others to recognise him as gay was lacking. The pandemic has changed the dynamic of the university workplace considerably and institutions are likely to continue some of the online practices adopted during lockdown longer term. However, for new colleagues to feel included in the university workplace, it is important that opportunities are created virtually as well as on campus, for all new staff to get acquainted with their colleagues and form positive professional and personal alliances. The response of this academic new to the university demonstrates the importance, highlighted by Ellis (2006) of signposting colleagues at induction to LGBTQ + staff networks and support services.

### Coming out to students

There has been much reflection on the personal, political and pedagogical benefits of LGBTQ + university staff ‘coming out’ to their students (Gates et. al, 2011). Despite the overall positivity about being out to colleagues, participants were far more cautious about being out to students. 20 colleagues stated they had not come out to students and were fearful of doing so. A cis gay female academic stated that she worried about students using her LBGTQ + status to undermine her, and worried her sexuality could be used as the basis of a spurious complaint if the students disliked her.

In the focus groups, several colleagues spoke of casual and pervasive homophobic language in classrooms. This took the form of students describing things that were negative as gay, such as ‘those trainers are gay’ or ‘this assignment is gay’ rather than being aimed at an individual (Nadal, 2013). This was acknowledged by staff as a legacy of language used widely by pupils in school and for some was not of great concern. However two lesbian lecturers agreed it shook their confidence and made them feel at the time quite unsafe in the classroom. Fric (2019) states that employees who conceal their sexual orientation are less likely to report discrimination but are more likely to perceive being discriminated against. No member of staff had reported the language, fearing that nothing would be done, or reasoning that the language was not intentionally homophobic.

Henderson (2019), quoting Markus and Nurius’s ‘possible selves’ (1986) recognises the complexity at the intersection of personal and professional teacher identities. She states that when educators do not feel able to come out to their students, the silent presence of that assumes heterosexuality which goes unchallenged. This was a matter of regret for one lesbian academic, who stated,

I don’t tell students about myself at all really. Which I which I regret…but life is just too complicated.

In *The Problem of Coming Out* (2004), Rasmussen interrogates the role model discourse and the potential conflict of the personal and professional values the teacher holds. Rasmussen observes that the pressure on educators to come out to students creates an identity half shaped by a heteronormative environment but balanced by the very exception to it. Henderson (2019) concurs, stating that the LGBTQ + teacher has two realities; to be the LGBTQ + role model they may have wanted when they were younger, versus being a ‘lonely flag bearer’ unable to function in the classroom at all. The role model discourse has the potential to erode the wellbeing of lecturers who cannot come out in class but may perceive that they have deserted those LGBTQ + students who need them. MacLure (1993) describes the identity of the classroom teacher as a ‘continuing site of struggle’ (p.313). It is also a working subjectivity that is formed and articulated through conversation, social interaction and self-presentation to students. Henderson (2019) states ‘This complex, cyclical matrix of past experience, present reflection, and future fears and aspirations works to constitute the present experience of the teacher’ (p.850).

In the focus groups, a frequent reason for not coming out to students in the classroom was the presence of some students of faith and sometimes, staff worried about their views towards LGBTQ + people. A cis gay male academic reported that when in one class, students were provided with an example of a same-sex couple in a case study, half a dozen students expressed disapproval, justifying their views with the phrase ‘it’s Adam and Eve, not Adam and Steve’. A cis lesbian academic too, noted religious and cultural beliefs as a tension in one or two of her classes.

LGBTQ + and faith identities are both are protected characteristics according to the 2010 Equality Act and tension may exist at the intersection of LGBTQ + and faith-based inclusive practices. There is extensive research in the US denoting the challenges LGBTQ + faculty staff face in Christian colleges (Coley, 2018; Scibetta, 2016). In 2019, when schools in England were first required to teach Relationships, Sex and Health Education (RSHE) inclusive of LGBTQ + relationships, some parents and representatives of faith communities in Birmingham and other major cities protested outside school gates in opposition. The strength of feeling was such that one teacher, who headed up a project called No Outsiders needed a police escort to and from his school at the height of the tensions (Khan, 2021). However, research by Valentine and Waite (2012) shows that in reality both LGBTQ + people and people of faith demonstrate an ethic of care towards marginalised others in recognition of their own complex intersectional identities. Whilst they acknowledge a potential geography of tensions between LGBTQ + people and people of faith, they observe in reality, that people of faith are willing to in day-to-day interactions to accommodate LGBTQ + people without changing their deeper held religious beliefs about LGBTQ + people abstractly. Of course, it is important to note that students of faith may themselves be LGBTQ + and in need of support.

### Signs and symbols of LGBTQ + identity

Those colleagues in the focus groups who did feel able to be out to students reported generally feeling more satisfied in their roles than those who were not out. This is echoed by Guasp and Balfour (2008) who found employees were more satisfied and felt a sense of loyalty to their employers when they perceived their workplace climates to be LGBT-supportive. None of the focus group participants had come out through a declarative statement to students, choosing instead to display signs and symbols of LGBTQ + inclusion. A cis lesbian senior lecturer explained that she had used her social media accounts and pride symbols to let students know she was LGBTQ + . She stated,

I was not particularly open to students for the first couple of years, but I started to be, after initially being open on Twitter and social media. Now I've now added the pride flag at my email signature, but I still haven’t said anything outright to my students.

Since coming out to students in this way, this lecturer reported becoming her faculty’s ‘LGBTQ + go to role model’. She reported having almost one student a month come out to her as trans and several LGBTQ + students regularly seeking her support for their wellbeing and mental health. Bird et. al., (2012) notes the importance of LGBTQ + affirmative role models for the health and wellbeing of LGBTQ + young people. This lecturer worried for these students during the pandemic, when lockdown forced many LGBTQ + students to return to their childhood bedrooms, often with parents who were not aware of their sexual or gender identity.

A cis gay male lecturer made a conscious decision to come out to students but also avoided a declarative statement in the classroom.

I put pride flag on my door because one of the things about being LGBT is it's a hidden or a non-visible characteristic but I wanted students to know I was there for them if they needed me.

Just as teaching staff had come out to students through the use of signals and symbols such as pride flags, or posts on social media, then the LGBTQ + staff surveyed acknowledged the contribution of LGBTQ + signs and symbols to their own feelings of safety in the workplace (Calvard et al., 2020). Even before he first arrived at the university, a cis gay male academic searched for clues that the university would be a safe workspace in which he could come out. Of note was the Stonewall Diversity Champion status of the university declared at the footer of documentation sent to him as part of his job offer and contract. This instantly made him feel at ease and had reassured him that he would be safe to bring his ‘whole self to work’. Whilst kite marks are sometimes are criticised for reducing inclusion to a series of tokenistic gestures (Prasad et al., 2011), this colleague recognised the power of them for indicating that a place is safe and welcoming.

Calvard et al. (2020) observe that symbols and signs of LGBTQ + identities in the workplace are imbued with nuanced narratives of power and control, and caution against the corporatisation of sexual identities at work. They observe that organisations gain social capital from signs and symbols that denote LGBTQ + inclusion. Universities often sponsor Pride parade floats, or arrange for land marks to be painted in rainbow stripes. However, the signs and symbols are empty if they do not have behind them an inclusion strategy which ensures LGBTQ + employees feel safe and included in their university workplace. Conversely, pride emojis or the inclusion of pronouns in an email signature can make an enormous difference to the lived experience of LGBTQ + colleagues and students. When they are accompanied by an inclusive culture, they can trouble the presumption of cis and hetero normativity and challenge oppressive workplace hierarchies.

One of the ways that LGBTQ + staff in universities can become intelligible to stakeholders is through personal presentation, especially dress (Hattrick, 2016). A cis lesbian working in professional services had initially used overtly feminine clothing to conceal her identity but fundamentally altered the way she dressed at work once she came out to her immediate team. She said,

I met somebody who said one of the most powerful things you can do as a lesbian is if you are butch then be butch. My appearance changed from that point on, there were no more dresses and heels, I cut my hair and wore only jeans and tee-shirts and this really helped me be myself at work.

When LGBTQ + employees in universities deviate from the expected gender appropriate corporate dress, in some small way they queer the university workplace. Lawler (2008) argues that clothing can be understood as ‘part of a wider social order that permits some actions and disallows others’. Butler describes this as ‘assisting a radical resignification of the symbolic domain, deviating the citational chain toward a more possible future to expand the very meaning of what counts as a valued and valuable body in this world’ (p.22).

A gay cis female academic wished for greater LGBTQ + representation at a senior level to give those not in leadership roles the permission and confidence to be themselves in the workplace. A gay woman in student-facing professional services concurred stating,

Visibility within senior staff is so important, as it enables all staff to feel that there is a path for them too, but this puts a lot of pressure on individuals to be visible and out at work.

The LGBTQ + participants in this study attributed the visibility of out LGBTQ + senior leaders to their feelings of safety at work. A gay male academic remembered seeing an overtly lesbian member of the Vice-Chancellor’s group for the first time, which led him to conclude,

OK, so we’ve got LGBT people in in high positions then so quite clearly being LGBT isn't a problem, because she has been promoted to the top or near the top.

Here, the visibility of a LGBTQ + colleague in a senior position led this academic to feel secure in his role and confident that progression to senior leadership was possible. Lee (2021), observes that when an LGBTQ + university leader is able to come out, it gives those who work for them permission to do the same. Role models in the workplace help individuals envisage their ideal or possible selves based on their own developing needs and goals (Gibson, 2004). Individuals observe workplace role models whom they perceive as similar in some dimension and actively observe and adapt attributes of people they perceive to be like them. However, the absence of diverse role models in senior positions can lead those with protected characteristics to believe that their progression in the university is not possible (Lee, 2021).

### Participant recommendations

In both the survey and focus groups, participants were asked to suggest what their university might do to foster greater LGBTQ + visibility. For the majority of participants, having access to specific LGBTQ + spaces on campus and online was paramount. Half the participants stressed the importance of a dynamic LGBTQ + staff network and stated that they would welcome the opportunity to join together with other diversity staff networks for joint conferences and events.

Participants also recommended line manager training in LGBTQ + inclusion, as some felt that they lacked the space or opportunity to speak to their manager or immediate teams about their personal lives. Too often, a culture of ‘don’t ask, don’t tell, denied them the opportunity to bring their entire selves into the university workplace and during the pandemic, working online made coming out extremely challenging for new staff.

In order to gain greater confidence to come out to students, participants advocated zero tolerance of casual and pervasive homophobia from students with all staff given the training in order to challenge inappropriate language when they heard it. Over half of the participants expressed a desire to see more LGBTQ + people in senior roles at the university and stated that mentorship or career development from an LGBTQ + leader or ally would be ideal, to help ensure LGBTQ + staff were promoted at the same rate as their heterosexual and cis gendered peers.

## Conclusion

This article has examined LGBTQ + staff attitudes to coming out in the university workplace and considered the part that signs and symbols of LGBTQ + inclusion play personally and institutionally. There are personal, political and pedagogical benefits to coming out in the university workplace, and it is widely seen as empowering for university staff. However, in the university workplace, sexual and gender identities are complex and structured in negotiation with peers, line-managers, senior leaders, students and other stakeholders. The university was shown to be an overwhelmingly welcoming work place for LGBTQ + staff, when compared to the other sectors staff had experienced, such as the NHS and uniformed services. The welcoming environment served to foster in those who were most confidently out amongst colleagues, a sense of loyalty and commitment long term to the university. However, the article demonstrates that LGBTQ + staff are a product of their previous experiences and in coming out to colleagues, younger members of staff showed greater confidence than those who had lived and worked in less tolerant times. The systems historical but enduring systems of power and knowledge in the workplace that traditionally sanctioned only identities and relationships that conformed to the norms and values of heterosexuality, male masculinity and female femininity, have left a damaging legacy for those who experienced them. Even within a university culture that is inclusive and welcoming, opportunities must be provided for LGBTQ + staff to speak about their personal lives and be authentic in the university workplace.

Coming out to students was a more challenging issue for the LGBTQ + staff. Student-facing staff were aware of casual and pervasive homophobia amongst students and felt that homophobic and transphobic viewpoints often go unchallenged. It is important that diversity training provides all staff with the confidence to challenging homophobic and transphobic language when it arises.

Signs and symbols of LGBTQ + identities such as rainbow flags, rainbow lanyards and LGBTQ + inclusive kite marks such as Stonewall Diversity Champion status is important to staff. Whilst some participants lacked the confidence to come out to students through a declarative statement, they used signs and symbols of LGBTQ + visibility to subtly indicate their identities to students. Signs and symbols of LGBTQ + diversity such as a rainbow flag on an office door or a pride emoji in an email signature, disrupted the cis and heteronormativity in the university workplace, creating LGBTQ + role models for those students seeking them.

Signs and symbols of LGBTQ + inclusion were also important to staff in reassuring them that their workplace was a safe space. As early as the interview stage for a role at the university, inclusive kite marks indicated to prospective employees that the university would be welcoming. Symbols that recognise LGBTQ + Pride and History months on campus also helped the staff to feel safe in the workplace, provided that inclusive policies and activities sat behind the gestures.

Finally, LGBTQ + role models are as important for LGBTQ + staff as they are for students, perhaps more so. When LGBTQ + staff are able to see LGBTQ + colleagues in positions of senior leadership, it helps them envisage their own career development and encourages loyalty and commitment to the institution.

This article describes a small scale study in a single institution and as such is able to make limited findings. Further research to examine the lived experience of LGBTQ + staff in the university. In particular research should focus on the inclusion and career pathways of professional services staff who have a distinct relationship to the university when compared to their academic peers.
